# Advances in Ureteral Stent Design and Materials

**DOI:** 10.1007/s11934-018-0779-y

**Published:** 2018-04-10

**Authors:** Ali Mosayyebi, Costantino Manes, Dario Carugo, Bhaskar K. Somani

**Affiliations:** 1 Bioengineering Science Research Group, Faculty of Engineering and the Environment, University of Southampton, Southampton, UK; 2 Institute for Life Sciences (IfLS), University of Southampton, Southampton, UK; 3 Department of Environment, Land and Infrastructure Engineering, Politecnico di Torino, Turin, Italy; 4 Department of Urology, University Hospital Southampton NHS Trust, Southampton, UK

**Keywords:** Ureteral stent, Stent encrustation, Biofilm, Material, Design, UTI

## Abstract

**Purpose of Review:**

There are three technological parameters that play a key role on the performance of an ideal stent. These are its material, design and surface coating. This article highlights some fundamental developments that took place in these three areas of stent’s technology, in order to contribute to the identification of an ideal stent.

**Recent Findings:**

In addition to technological developments concerning stent’s material, design and surface coating, the flow dynamic performance of stents has recently attracted increasing attention. Notably, it has been postulated that the local flow field in a stent is correlated with the deposition of crystals and microorganisms. These findings could potentially revolutionise future stent’s designs, and complement developments made on materials and coatings.

**Summary:**

The most relevant changes in materials, designs and surface coatings of ureteric stents are reviewed in this article. These are described in the context of a specific cause of stent’s failure they aim to address, with a particular focus on encrustation and biofilm formation.

## Introduction

During the last few decades, ureteral stents have been widely utilised as a measure for temporary or permanent drainage for the occluded upper urinary tract. The underlying rationale is to allow the urinary flow to bypass internal or external obstructions, which impair its drainage.

Nonetheless, there are few side effects associated with stents that complicate their use and management, particularly when deployed as a long-term treatment option. Bacterial colonisation and encrustation [1–3] over the stent surface are two of the most common causes of stent-related infections and obstruction, potentially resulting in its functional failure. Given that these issues profoundly affect the therapeutic outcome, patient’s quality of life and associated costs for healthcare providers, further efforts should be put in place to address them effectively [4, 5•, 6–9].

## Materials and Methods

In this paper, the main functional properties of stents are reviewed, including their architecture, operating principle, constitutive material and surface coating. Side effects and complications associated with ureteral stenting are subsequently discussed, with particular emphasis on particle deposition (i.e. as a leading cause of encrustation and biofilm formation).

In order to identify publications relevant to ureteral stent development for this review paper, we conducted a PubMed search from 1970 to 2017. The keywords more extensively used for the search were “ureteral stents”, “stent encrustation”, “stent biofilm”, “stent bacterial colonisation”, “stent design”, “stent material”, “stent coating” and “UTI”.

## Historical Perspective

According to Bareeq et al. [10], the history of ureteral stents goes back to ancient Egypt. The first procedure of ureteral catheterization, which involved insertion of a tube inside the urinary system via open bladder surgery, was performed by Gustav Simon in 1900 [11]. The first ureteral stent with an architecture comparable to the ones currently used was introduced by Joaquin Albarrano in the early 1900s [10]. Since then, technological advancements have had a significant impact on the design and material properties of stents. Each of these developments aimed to address a specific failure or cause of morbidity. In this review article, they are discussed, together with the issues they attempted to resolve.

## Constitutive Materials

Over the last few years, engineers and scientists have worked on identifying optimal constitutive materials for ureteral stents, focusing specifically on mechanical strength, flexibility, biocompatibility, surface roughness and cost-effectiveness.

There are two main types of biocompatible materials [12–14] that are generally used for fabricating ureteral stents: polymers and metals.

The first ureteral stents were constituted of polyethylene, a synthetic polymer [15]. However, rigidity and tendency to break limited their usage in the clinical setting. To overcome these limitations, Gorman et al. [16] introduced a mixture of polyethylene and polyurethane as a more resistant material option against encrustation.

Silicone is widely employed for manufacturing ureteral stents, thanks to its flexibility against bending and lubricious properties. However, the rigidity of this material can potentially become a disadvantage during the insertion of the stent within a guidewire [17, 18].

Different polymeric materials also present a variable tendency to encrustation. A study performed by Tunney et al. [17] compared stents made of five different polymeric materials against encrustation, over a period of 14 weeks of suspension in an artificial urine. Notably, due to its smoother surface, silicon appeared to have the best performance in the long term, showing 30% less encrustation at 10 weeks. Moreover, this study demonstrated that silicone was the least prone to calcium deposition [17].

Limited work has been conducted to test the effect of constitutive materials on the mechanical strength of the stent. For instance, Hendlin et al. investigated the strength and rigidity of 12 commercially available stents, before and after exposure to artificial urine (30 days) in static conditions [19]. They evaluated coil strength (defined as the maximal force required to pull the proximal coil through an artificial tissue) and rigidity (Young’s modulus). The stiffest and softest stents were Cook® C-Flex (a copolymer from the silicone family) and Cook® Black Silicone, respectively. Moreover, Applied Vertex® and Cook® Endo-Soft AQ had the highest and lowest coil strength, respectively.

Christman et al. [20] also compared the radial compression of different stents and concluded that wire reinforced stents, such as resonant stents, can withstand greater compression (i.e. such as the one caused by malignant urinary tract compression), without a significant reduction of the stent’s inner lumen.

There have been few studies evaluating the effect of stent’s rigidity on patients’ quality of life. Some of these studies, such as those by Bregg et al. [21], Pryor et al. [22] and Joshi et al. [23], have demonstrated almost no correlation between patients’ quality of life and the material composition of the stent. On the other hand, a study by Lennon et al. [24] on 155 patients revealed that the softness of the stent has a direct influence on patients’ tolerability, and that softer materials were associated with higher incidence of dysuria and pain [25, 26].

Metallic stents were introduced by Gort et al. [27]. Their purpose was to reduce stent-associated morbidity, and increase their ability to oppose deformation caused by extrinsic/intrinsic ureteric obstruction. However, in an in vivo study on 50 patients by Liatsikos et al. in 2009, metallic stents (specifically Resonant® metallic stent by Cook® medical) suffered from encrustation, as observed upon removal, and therefore did not provide a significant reduction in encrustation rates [28].

Kirby et al. performed a study on 30 patients to examine the performance of a titanium stent as a treatment modality for bladder blockade due to benign prostatic hyperplasia (BPH) [29]. Their results showed effective urinary flow re-establishment in 25 patients. In another study, Song et al. employed an expandable stent made by Nitinol (nickel-titanium) with the purpose of managing urethral strictures [30] and the result demonstrated the viability of nitinol stent in urethral strictures’ treatment.

The current metallic stents are made of nickel/titanium mixed alloys. These materials have a specific memory that allows them to soften at temperatures below 7–13 °C, and retrieve their shape upon increasing the temperature above 55 °C. This property is highly advantageous in the process of stent deployment and removal [31].

## Stent Design

‘Double-J’ refers to the most common type of stent design that was initially introduced by Finney in 1978 [32]. The term ‘double-J’ refers to the ‘J’ shape of each end of the stent, which is designed to anchor the stent and prevent its displacement. Since then, different biomedical companies have fabricated stents that have different architectures with the main aim of decreasing the impact of encrustation and infection, as well as improving urine drainage and lessen the impact on patients’ quality of life. Some of the most commonly used designs are reported and discussed in the following paragraphs [10, 33].

*Grooved* stents, having external grooves along the stent lumen, were introduced by Finney in 1981 [34]. This design was developed specifically as a post-lithotripsy treatment option, in order to improve the stone clearance by introducing multiple pathways for urine drainage [35]. Grooved stents have been manufactured by Olympus® (USA) under the name of LithoStent™.

*Spiral* stents were initially introduced by Anderson et al. in 1987 [36]. This design had a metal wire within the stent to maintain it into a spiral shape, and was believed to improve urine drainage in case of extrinsic blockage by providing a stable and durable opening of the ureteric lumen. In 2000, Stoller et al. employed the spiral design to evaluate urine flow in an in vitro model. Results from this study demonstrated increased flow in the model using a spiral stent as opposed to the traditional design (Double-J stent with straight lumen) [37]. The spiral shape was then improved to the next generation called *spiral cut*, where the stent itself looked like a tube while its wall had a spiral cut. An in vivo study on 12 swines performed by Mucksavage et al., however, found no statistically significant difference between spiral cut and other stent models, in terms of encrustation rate, infection or stent migration. The spiral design demonstrated superior ability to conform to the ureter shape [38]. Percuflex Helical™ (Boston® scientific, USA) represents a commercially available spiral stent model.

*Self-expanding meshed* ureteral stents were developed with the aim of decreasing the irritation of the urinary tract and increasing urine flow within the stent, by taking advantage of the meshed structure to reduce the likelihood of clogging [26, 39]. A few studies assessed the performance of this design. For instance, the work of Olweny et al. revealed increased flow through the stent in comparison to traditional stents, which also reduced reflux towards upper tract and flank pain [40].

Other advantages associated with meshed stents are their limited influence on the mechanical properties (i.e. distensibility) of the ureter, and potential for elution of bioactive compounds using the mesh structure as a drug reservoir [41]. Drug-eluting mesh stents have been reported, i.e. for delivery of anticancer drugs (paclitaxel) [42] or anti-inflammatory agents. Examples of this application can be found in the work of Lugmayr and Pauer (1992), Barbalias, Siablis et al. (1997), Burt and Hunter (2006) and Wang and Burgess (2010) [43–45].

Despite the aforementioned advantages, mesh stents suffer from complex insertion procedures [40] and higher cost [10].

*Tail* stents are very similar to the traditional double-J stent. The main architectural difference is at the distal end of the stent, where there are loops of polymer instead of the classical pigtail. The rationale behind this design was to decrease the bladder irritation that the standard stents caused. A randomised study on 60 patients performed by Dunn et al. revealed lower levels of irritation and obstructive urinary symptoms by using this stent compared to a traditional pigtail stent. However, incidence of flank or renal symptoms did not show a significant difference between the two types of stent [46]. Yew et al. [47] demonstrated that stent insertion and removal caused minimal pain on patients treated with tail stent. Companies have employed different materials in manufacturing tail stents, with the aim of improving patients’ acceptability and comfort [48]. However, the work of Davenport et al. [49] using two different stent models, namely Inlay® (Bard® medical, USA) and Polaris™ (Boston® scientific, USA), demonstrated that there was no significant difference using either of these stents in 98 patients.

*Dual-durometer* stents have a similar architecture to that of tail stents. The main difference is in the mechanical properties of the stent body, which transitions from harder at the proximal end (kidney) to softer at the distal end (bladder). This design was introduced with the purpose of decreasing irritation due to its soft composite tail, and therefore increasing the tolerability of the stent [10]. Boston® Scientific (USA) has employed this architecture in the Percuflex® ureteral stent series.

*Magnetic-tipped* stent was first introduced by Maculuso et al. in 1989 [10, 33], and was developed mainly to decrease additional costs associated with stent removal. Based on a study by Netto et al. in 1997–2000, this design had the potential for decreasing removal-associated costs by over £1000 per patient [50]. Taylor et al. presented a more recent version of this design that confirmed successful removal of the stent in 29 of 30 patients [51]. This type of stent design also does not require cystoscopy for stent removal.

Modifications of the previously discussed stent designs have led to other types of ureteral stents. One example is the so-called dual lumen stent, which has two drainage pathways to provide compensation in case of stent obstruction. This design was tested in an ex vivo kidney model by J. Hafron et al. [52] who reported improved urine drainage over time compared to single lumen stents.

Another, more recent design is the resonance metallic stent [53], which consists of a compressed spring without side holes, introduced by Cook© Medical with the aim of indwelling stents lasting up to 12 months or longer. Although the upfront cost of this stent is higher compared to polymeric stents, the overall economic impact becomes less significant considering that polymeric stents often require removal and replacement. Moreover, initial tests performed by Wah et al. [54] on 15 patients showed improved urine drainage in comparison to traditional double-J ureteral stents, over a period of 1 year.

## Complications and Side Effects Associated with Ureteral Stenting

Placement of ureteral stent sometimes leads to localised inflammation, which can cause haematuria and bladder pain. Elevated bladder pressure can potentially cause pain due to urine reflux towards the kidney. Abdominal and intestinal pain may be associated with these undesired side effects [55, 56]. Insertion of a stent can also cause stone retropulsion towards the kidney [37, 57, 58]. It has also been demonstrated that ureteral stents have a direct effect on ureteral peristalsis, which in turn impacts on urine flow and kidney pressure, thereby increasing patients’ discomfort during urination. Reduced ureteric peristalsis has also been shown to cause renal pelvis inflammation [37, 59–61]. Bladder irritation has been associated with stenting, and may cause urinary urgency and other urinary symptoms [62, 63].

Stent migration from its primary site is recognised to be another major complication. However, its occurrence has been significantly reduced since the introduction of ‘J ends’ (or pigtail ends), which have an anchoring effect minimising stent’s displacement over time. In addition, polyurethane is recognised to have better shape memory (and thus to more effectively conform to the urinary tract) compared to silicone, reducing the likelihood of ureteral stent migration along the urinary tract. On the other hand, stents made by softer materials have been found to be more prone to migration [56, 64–66].

Since ureteral stents are foreign to the urinary system, their surfaces create an environment for colonisation by bacteria that may potentially form biofilms. This complication could lead to premature removal or replacement of the ureteral stent. For example, in a study performed by Paick et al. [67] using Percuflex® (Boston® Scientific, USA) stents, bacterial colonisation occurred in about half of 52 patients, after 2 weeks from stent insertion.

Encrustation is another complication that may affect indwelling of ureteral stents. It occurs in association with the presence of bacteria (such as *Proteus mirabilis*), which are known to produce urease. These bacteria cause an increase of urine pH, leading to crystals’ formation [68•, 69]. There are different factors that could affect stent encrustation, such as urine composition and pH, stent’s material and surface properties, stent dwell time and urine flow dynamics [65]. These problems are discussed further in the following sections.

## Current Approaches to Minimise Encrustation and Biofilm Formation

Different stent materials can cause variable levels of encrustations. Some of the common metallic alloys that have been used for producing ureteral stents (i.e. nitinol, superalloy titanium and chromium cobalt) have shown different tendency to encrustations [70, 71]. A study by Tunney et al. [17] demonstrated that silicone and polyurethane had higher resistance to encrustation compared to other materials, after 2 weeks of stent insertion. At 10 weeks from insertion, silicone started to show superior performance than polyurethane.

Coating of the stent surface has been explored as a modality to decrease or ideally impede encrustation or biofilm formation over the stent surface [72•]. This provides a convenient route for the industry, which may enable the use of conventional materials for constructing the stent, followed by coating of their surface [33].

For instance, some of the coatings employed on polymeric stents increase surface smoothness, improve biological tolerability and lower encrustation over longer time periods (up to 1 year) [73]. As an example, glycosaminoglycans (CAGs) [74, 75]—that are a component of urine—are employed as natural coating to prevent encrustation [76]. Heparin, a highly sulfated member of CAGs family and a blood thinner, has demonstrated potential for delaying surface encrustation for up to 12 months in a study performed on patients by Cauda et al. [77]. Watterson et al. employed stents coated with oxalate-degrading enzymes and showed reduced stent encrustation in 40 New Zealand white rabbits [78].

Diamond-like carbon (DLC) has also been used for coating stents, because of their physical and chemical properties [79]. Laube et al. performed a study on 10 patients (average duration of 14 weeks) and demonstrated the capability of DLC coating in reducing rates of encrustation and biofilm formation, resulting in improved stent’s lifetime [80].

Hydrogel coatings are used due to their hydrophilic porous structure that allows forming a thin hydrated layer over the stent surface. This layer was claimed to prevent biofilm growth, as it counteracts the formation of the conditioning layer often indicated as a primary cause of biofilm formation. However, a study performed by John et al. [81] showed that hydrogel coating on its own does not reduce bacterial adhesion, but instead performs better when it is combined with other chemicals, such as antibiotics. Their study included comparison of hydrogel-coated and uncoated stents dipped in antibiotic solutions, which were both suspended in *Escherichia coli* or *Enterococcus faecalis* solutions for 24 h.

Phosphoryl-choline (PC), which is an essential component of erythrocytes aping lipid membrane, provides a hydrophilic environment that hosts water and protects the surface of the stent from proteins or other chemicals [82, 83]. The use of this coating has demonstrated a slight reduction in encrustation and biofilm formation, as shown by Stickler et al. in a clinical study on 44 patients over the period of 12 weeks [82]. Encrustation and biofilms were analysed using scanning electron microscopy in both coated and uncoated stents.

Antibiotic coating can either prevent growth of bacteria or kill them. It has been performed widely by either dipping the stent inside a solvent containing antibiotics and evaporating the solvent, or incorporating the antibiotics into biodegradable coatings for sustained release [84]. There are different types of antibiotics that have been used in stents, such as daptomycin, linezolid, tigecycline, rifampicin [85, 86], temporin A, RNAIII-inhibiting peptide [87], oxacillin, cefotaxime and vancomycin [88]. Each of these was tested in different in vivo and in vitro models and demonstrated a reduction in bacterial growth. However, it has also been demonstrated that a combination of antibiotics often has a stronger effect against bacteria. For instance, Minardi et al. [89] demonstrated such effect by deploying the combination of *tigecycline* and *rifampin* in 5 rat models against *Enterococcus faecalis*.

Polytetrafluoroethylene (PTFE), also known as Teflon™, is a material acknowledged for its low friction coefficient and resistance against Van der Waals forces, both factors that affect bacterial colonisation. This makes PTFE a promising candidate for stent’s coating [90•]. Chung et al. [91] compared 14 PTFE coated vs uncoated metallic mesh stents in 7 dog models, over an average period of 15 weeks, and investigated the effectiveness of PTFE coating against tissue ingrowth, and found the coating to be effective against luminal occlusion caused by it.

Coating with antimicrobial agents is another strategy to reduce encrustation and/or biofilm formation. Two examples of such agents are triclosan and silver. A recent study on the use of triclosan coating has been performed by Lange et al. [92], comparing coated vs uncoated stents incubated in stationary conditions for 7 days. It confirmed the significant bacterial resistance of this agent. Despite offering resistance to biofilm formation, triclosan did not get FDA (Food and Drug Administration) approval due to concerns over its potential for developing antibiotic resistance [84]. On the other hand, silver ions have shown to be capable of preventing bacterial replication [93]; however, their performance against biofilm formation in ureteral stents has been variable [87]. Additionally, prolonged use of the coating could lead to argyria [84].

Chitosan is a non-toxic biopolymer that has also demonstrated potential for inhibiting bacterial growth. Its anti-biofilm properties are shown by Carlos et al. [94], who tested this specific coating against bacteria over 54 h, using a drip-flow biofilm reactor system. In another study, Yang et al. [95] combined chitosan and polyvinylalcohol (PVA) to successfully reduce the absorption of proteins and prevent bacterial growth in ureteral catheters made by segmented polyurethane in a static study.

### The Role of Flow Dynamics on Stents’ Failure

It has been demonstrated that urine flow dynamics in a stented ureter has an important role in governing the formation and growth of encrusting particles and bacterial deposits. This relationship was investigated theoretically by Siggers et al. [96] and Waters et al. [97] suggesting a strong relation between the local flow dynamics and the deposition and growth of encrusting crystals. Using an artificial model of the stented ureter, Clavica et al. observed the formation of vortices in the vicinity of a ureteric obstruction, which trap particles suspended in the fluid promoting their deposition [58, 98]. More recently, we have used computational and experimental models and demonstrated a strong correlation between accumulation of particles and wall shear stress in ureteric stents [99••, 100••]. These findings could open new avenues for improving the stent’s design via fluid dynamic optimisation, and provide technological solutions that are complementary to materials and surface coatings.

## Conclusion

Various advancements in materials, design and coating of ureteral stents are reviewed in this paper and summarised in Table 1. Each of these developments aims to address specific causes of stent’s failure, especially encrustation and biofilm formation. Combining the most appropriate material, design and coating would allow the development of an optimal stent.

**Table 1:** Different stent materials, designs and coatings with few key aspects of each and where relevant (especially in the design section) an example of a commercial stent is provided

	The change	The solution	Reference
Material	Polyurethane	Better drainage efficiency compared to silicone	In vitro model made by 9F polyvinyl tubing and ex vivo model taken from a human cadavericurinary system [101]
	Silicone	Better performance against encrustation compared to polyurethane	Ureteric stents were suspended for 15 weeks in artificial urine and the amount of encrustation was measured using atomic absorption spectroscopy [17]
	C-Flex	A thermoplast polymer from the family of silicones. Its surface of friction was lower compared to polyurethane and Percuflex™	Various stent materials’ surface coefficient of friction were measured by Mardis et al. [102••]
	Percuflex™	From the family of silicones. A biomaterial with a relatively long-term indwelling biodurability compared to polyurethane and silicone itself.	
	Titanium	Improvement in benign prostatic hyperplasia in over 80% of patients	The performance of titanium stent was investigated on 30 patients in a study carried out by Kirby et al. [29]
	Nitinol	A mixture of nickel and titanium that softens at temperatures below 10 °C and hardens as the temperature increases and allows better stent insertion and removal	The performance of 22 nitinol stents was tested in 12 male patients between 19 and 67 years old [30]
	Stainless steel	Does not have major insertion side effects andrecognised as an operational tool in tumour associated hydronephrosis	23 patients (29 to 78 years old) with hydronephrosis being the main pathology [43]
Design	Grooves	Providing multiple pathways for urine drainage	LithoStent™ (Olympus®, USA)
	Spiral	Providing a stable and durable lumen	Percuflex Helical™ (Boston® scientific, USA)
	Self-expanding	Providing a wider pathway for urine compared to conventional stents	UVENTA™ (TaeWoong ®, South Korea)
	Tail	Provides less bladder irritation compared to the conventional stents	Inlay® (Bard® medical, USA) and Polaris™ (Boston® scientific, USA)
	Dual-durometer	Provides less bladder irritation compared to conventional stents and better stability in the kidney	Percuflex® (Boston® scientific, USA)
	Magnetic-tipped	Provides an improvement towards stent removal and avoiding the use of cystoscopy	Magnetic Black-Star (Urovision, Germany)
	Resonant	Provides up to 12 months indwelling	Resonant ® (Cook® medical, USA)
Coating	CAGs and heparin	A natural component of urine that could potentially delay encrustation for up to 12 months	40 rabbits were tested over a 30 days period and encrustation was measured using atomic absorption spectroscopy [78]
	DLC	With physical and chemical composition reducing encrustation and biofilm formation	10 patients were treated for the period of 14 weeks [80]
	Hydrogel	Preventing biofilm via creating a thin layer of water on the surface	Coated and uncoated stents were suspended in bacterial solvents for 24 h [81]
	PC	A natural component that provides a hydrophilic environment on the surface and as a result reduces encrustation and biofilm formation	Coated and uncoated stents were tested on 44 patients for a period of 12 weeks [82]
	Antibiotic	Disrupts bacteria formation and growth	Studied on 5 rat models against *Enterococcus faecalis* [89]
	PTFE	Has a low friction coefficient and resistance against van der Waals forces that prevents bacterial colonisation	This was studied by comparing coated vs uncoated 14 metallic mesh stents in 7 dog models for the period of 15 weeks [91]
	Antimicrobial triclosan and silver	Triclosan has a significant bacterial resistance however it is not approved by FDA, because of concerns over antimicrobial resistance. Despite resistance against biofilms, a prolonged use could lead to argyria.	Coated and uncoated stents were investigated for a period of 7 days in a stationary study [92]
	Chitosan	It inhibits biofilm formation on the stent surface	Coated surface was exposed to different bacteria through a drip-flow biofilm reactor system for a period of 54 h [95]

Moreover, urine flow dynamics plays a crucial role in governing encrustation and biofilm formation in stents, and it should be also considered in the development of a stent’s design. Currently, there is no ideal stent that does not experience complications and failures. However, authors hope that this review provides a summary of the technological challenges that we need to overcome for developing a better stent.

## Abbreviations

CAGs: Glycosaminoglycans
DLC: Diamond-like carbon
PC: Phosphoryl-choline
PTFE: Polytetrafluoroethylene
PVA: Polyvinylalcohol
UTI: Urinary tract infection
